# Simulation Interventions for the Classroom to Support the Acquisition of Interprofessional Competencies

**DOI:** 10.7759/cureus.14662

**Published:** 2021-04-24

**Authors:** Brenda J Gamble, Leslie Graham, Helene-Marie Goulding, Evelyn Moreau, Brenda Barth

**Affiliations:** 1 Medical Education and Simulation, Faculty of Health Sciences, Ontario Tech University, Oshawa, CAN; 2 Medical Education and Simulation, School of Health and Community Services, Durham College, Oshawa, CAN; 3 Health Sciences, Ontario Tech University, Oshawa, CAN

**Keywords:** interprofessional education, competency-based learning, interprofessional collaboration, interprofessional simulation

## Abstract

Interprofessional collaboration (IPC) supports the delivery of quality and safe healthcare. The acquisition of interprofessional competencies both pre-licensure and post-licensure are key to implementing this approach in the healthcare workplace. This report documents the development and implementation of a simulation intervention to support interprofessional education (IPE) in the undergraduate classroom for pre-licensure learners. The learning activity framework includes an exposure phase (e.g., didactic classroom instruction) and an immersion phase (e.g., simulation intervention and debriefing). Details on the debriefing process are included as it is key to achieving the learning objectives. The three learning activity pilot tests (n=150) revealed that learners recognized that interprofessional competencies were an important asset to support IPC. The pilot tests identified the need for further development in order for students to make a connection with the mastery phase (i.e., clinical placement). The next steps will include the development and incorporation of formative tools to assess learners’ progress, as well as a plan to evaluate the learning activity that will connect all three phases (exposure, immersion, and mastery) of the learning framework.

## Introduction

Providing quality and safe healthcare is a priority both in Canada and internationally [1]. Interprofessional collaboration (IPC) is one of many strategies identified to support these priorities [2]. The implementation of IPC is dependent upon the establishment and maintenance of partnerships between different health and social care workers, and patients [3]. However, a number of challenges exist within the healthcare labor force that hinder the successful implementation of IPC [e.g., differing attitudes about working collaboratively in interprofessional teams (IPTs), established stereotypes about health and social care workers] [4]. Interprofessional education (IPE), both pre-licensure and post-licensure, is viewed as a way to enhance understanding and respect for other workers’ expertise by developing communication and interpersonal skills to work collaboratively [5]. IPE is a pedagogical approach where, “two or more professions learn with, from and about each other to improve collaboration and the quality of care” [6]. Providing opportunities for health and social care workers to learn together can prove challenging due to a number of factors including discipline-specific curricula requirements, certification requirements, scheduling logistics, and the availability of human and financial resources [7]. At the same time, healthcare education and training are transitioning from structure- and process-based education to competency-based education [8].

In light of these challenges, an IPT of educators in the Faculty of Health Sciences at Ontario Tech University (Ontario Tech) developed and produced four simulation interventions to support IPE in the classroom for the learners (i.e., pre-licensure) enrolled in the Medical Laboratory Science and Public Health Science programs at Ontario Tech and the Nursing Collaborative program offered by both Ontario Tech and Durham College in Oshawa, Ontario. The learning activities created and implemented focused on the development of the interprofessional competencies of role clarification, team functioning, collaborative leadership, interprofessional communication, interprofessional conflict resolution, and patient/client/family/community-centered care to support the implementation of IPC [9,10].

We report on the process undertaken to develop and create the four standalone simulation interventions. Content for each of the four interventions incorporated at least two or more IPC competencies. This report provides details on the simulation intervention titled “Interprofessional Collaboration with Infectious Disease Control”. The learning activity reported here focuses on interprofessional communication to describe the activity more fully and to report on the feedback from learners.

## Technical report

Conceptualization of the learning activity

**Figure 1:**
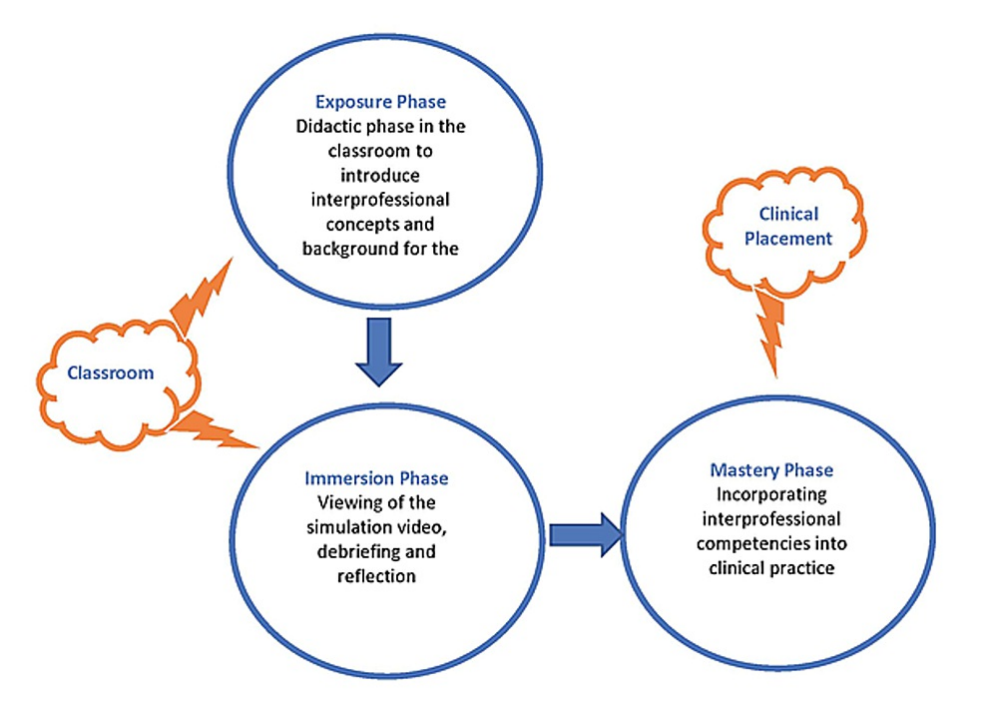
Charles, Bainbridge, and Gilbert framework

Different models of IPE exist, including didactic programs, case studies, community-based experiences, and interprofessional simulation interventions [11]. The model that was used to develop the learning activity for the classroom in this report was based on the integration of a didactic process and a simulation intervention (i.e., video). The learning activity was developed for new learners as well as for continuing education. IPE is a life-long process as IPT memberships change in response to the evolving and different needs of patients and the location of the delivery of care (e.g., hospital, home, community). This process of learning is captured by the framework established by Charles, Bainbridge, and Gilbert [12]. The framework is comprised of three phases (Figure 1).

Our goal was to develop a learning activity that incorporated both the exposure and immersion phases for the classroom across all programs (i.e., medical laboratory science, collaborative nursing, and public health programs). The purpose of the learning activity was to prepare learners for their clinical placements or the mastery phase of the learning process. The exposure phase, using a didactic process, was designed to introduce the learners to the concepts of IPC, IPC competencies, and IPE as well as the challenges and benefits of each concept. Next, the simulation interventions were then used to engage learners in the immersion phase.

Simulation interventions

An integrative approach was taken to identify the content for the simulation interventions. This included a scoping review of the peer-review literature and identification of cases based on the experiences of health educators and practitioners. The simulation experience was guided by the International Nursing Association for Clinical Simulation in Learning (INACSL)’s Standards of Best Practice: Simulation (2016) in all aspects of design and delivery [13]. Once the content was developed, it was reviewed and validated by team members who are subject matter experts in their respective fields. Table 1 provides the details on the simulation intervention highlighted in this report. The learning activity focused on the IPC competency of interprofessional communication.

**Table 1:** Overview of interprofessional communication simulation intervention

Communication simulation description	A father and mother bring their small child to the local emergency department. The child is examined and is found to have a high fever. The attending physician orders laboratory tests. The test results are delayed and the child’s condition deteriorates. The laboratory cannot complete the laboratory tests due to mislabelling of the specimens. There is no communication between the emergency department and the laboratory until the attending physician queries about the laboratory test results

Initially, the learning objectives were developed (Table 2), and then storyboards were developed to provide contextual details to guide the development of the IPC simulation interventions. The Readiness for Interprofessional Learning Scale (RIPLS) questionnaire [14] was incorporated into the learning activity as a pre- and post-test during the exposure and immersion phases respectively to determine the progression of learners’ attitudes on IPE.

**Table 2:** Learning objectives

The learner will be able to:
List the five competencies of interprofessional collaboration
Describe the importance of effective interprofessional communication with other healthcare professionals
Recognize the need to listen to others/team members
Discuss the importance of communication at the beginning and end of shifts (handover or handoffs)

The team recruited student volunteers to fulfill the roles of simulated patients and healthcare workers as depicted in the simulation interventions. The Collaborative Nursing program’s satellite simulation laboratory and the Medical Laboratory Science program’s hands-on laboratory were used to set the background for the video-recorded scenes. The filming and production of the simulation interventions were completed by Ontario Tech’s Teaching and Learning Centre. Video 1 illustrates the interprofessional communication simulation intervention.

**Video 1:** Simulated interventions for interprofessional education

Debriefing

The simulation intervention is only one part of the immersion phase, and additional elements are needed to enhance understanding and encourage reflection on the simulated experience [15]. After the learners had viewed the simulation intervention, the next step in the learning activity was the debriefing session. The following debriefing questions were used to structure this learning experience: 1) How did this simulation make you feel? 2) What do you think was happening? 3) What would be an example? 4) Why did this occur? 5) What was the purpose of the simulation experience? 6) How can this simulation help in real-life practice? and 7) What were the take-home messages of the simulation?

The type and content of the questions can vary depending on the learning goals of the learning activity. The debriefing part of the learning experience was overseen by a facilitator responsible for keeping the debrief focused on the learning objectives [16]. A facilitator who was not the instructor of the course led the debriefing and recorded the comments provided by the students. The recorded notes were used to summarize the discussion after the debriefing.

## Discussion

Debriefing proved to be an important part of the learning process, as learners initially focused on the psychomotor skills illustrated in the simulation intervention. For example, a course instructor who was also a manager in a hospital-based laboratory pointed out that the scenario depicted in the simulation would not have occurred in their workplace. The course instructor was focused on the healthcare workers' technical proficiency (e.g., following appropriate analytical procedures). It was the role of the facilitator to acknowledge the point made by the course instructor and to then refocus the discussion on the learning objectives. The focus of the learning activity was on the acquisition of affective skills associated with interprofessional communication. The development of the scenario was based on a real-life case with one exception. Unlike the outcome in the simulation, the child died in real life. It was determined that one of the contributing factors to the child's death was the lack of effective interprofessional communication. The reason for not including the death of the child in the simulation was to not distract the learners from the learning objectives of the learning activity. As a result, the child in the simulation fully recovered. 

Further discussions after the debriefing reflected on the realization that factors associated with providing quality and safe healthcare required a system-oriented approach rather than a person-centered approach (i.e., blaming individual healthcare workers). This is a view supported by The Institute of Medicines’ report titled “To Err is Human: Building a Safer Health System” [17]. The education, training, skills, and actions of healthcare workers are all factors paramount to ensuring quality and safe healthcare. This key insight emerged as learners reflected on the experience in terms of providing quality and safe healthcare.

## Conclusions

Addressing the challenges faced by today’s health systems will require health professional educational programs to include competencies that support IPC into the curricula. However, recognizing the potential impact of IPC is only the first step. Incorporating IPE into the curricula can be a daunting task for educators who are faced with the profusion of new clinical knowledge. Educational programs are also faced with the challenge of how best to prepare students for clinical placements. Uni-professional educational programs often lack the ability to deliver IPE due to a number of reasons (e.g., lack of recourses, scheduling logistics). Hence, simulation is a tool that can be used to implement IPC competencies into the classroom to support IPE.

Working in a team comprising educators from different health professional programs to create IPE activities can be challenging (e.g., coordination of different schedules and logistics). However, upon reflection of the experience to create the learning activities, the benefits far outweighed the challenges. All team members were champions of IPE and were able to build on each other’s strengths and expertise to work collaboratively. In fact, many of the team members have since collaborated with other faculty members from both Ontario Tech University and Durham College to develop new IPE learning activities using simulation. Financial resources were not a barrier as we received institutional support from Ontario Tech’s Innovation Teaching Fund.

The students’ contribution during the filming of the simulation intervention, the pilot testing, and the invaluable feedback they provided throughout the entire process were key to the project’s success. Students who participated in the pilot tests acknowledged that the acquisition and awareness of interprofessional competencies were necessary to support IPC. The IPE simulation provided a safe environment (e.g., free from performance assessment during clinical training) for learners to reflect upon the importance of effective interprofessional communication. However, the pertinent question is this: will this better prepare the learner for clinical placement (i.e., the mastery phase)? The learning activity needs to be developed further to make a connection with the mastery phase. Further work needs to be done to incorporate formative evaluations into the learning activity throughout the three phases to demonstrate learner progress. Additionally, a plan to evaluate the learning activity that will connect all three phases of the learning framework needs to be devised.
